# A prospective study on the effect of self-reported health and leisure time physical activity on mortality among an ageing population: results from the Tromsø study

**DOI:** 10.1186/s12889-020-08681-x

**Published:** 2020-04-28

**Authors:** Ida Marie Opdal, Lill Sverresdatter Larsen, Laila Arnesdatter Hopstock, Henrik Schirmer, Geir Fagerjord Lorem

**Affiliations:** 1 Department of Psychology, Faculty of Health Sciences, UiT - The Arctic University of Norway, Tromsø, Norway; 2 Department of Health and Care Sciences, Faculty of Health Sciences, UiT - The Arctic University of Norway, Tromsø, Norway; 3 Department of Community Medicine, Faculty of Health Sciences, UiT - The Arctic University of Norway, Tromsø, Norway; 4 Department of Clinical Medicine, Faculty of Health Sciences, UiT - The Arctic University of Norway, Tromsø, Norway; 5 Department of Cardiology, Akershus University Hospital, Lørenskog, Norway; 6 Department of Clinical Medicine, Faculty of Medicine, University of Oslo, Oslo, Norway

**Keywords:** Ageing, Cohort studies, Physical activity, Self-reported health, Mortality, Longitudinal study

## Abstract

**Background:**

The prevailing Western ideal of ageing in place, with the option to stay at home as one ages, has led to the development of physical activity guidelines for people of advanced age to increase their quality of life and promote their functional abilities. This study investigates the effect of self-reported health and physical activity on mortality and examines how levels of age-specific physical activity affect self-reported health trajectories in an ageing cohort.

**Methods:**

The sample cohort of the population-based Tromsø Study consists of 24,309 participants aged 25–97 years at baseline. This study involved a survival analysis from 1994 to 2015 and included those who completed two or more surveys (*n* = 12,241) between 1994 and 2008. The purpose was to examine the relationship between physical activity and self-reported health throughout life using a random coefficient model analysis.

**Results:**

Being sedentary was associated with an increased risk of mortality in the ageing cohort. Subjects who reported neither light physical activity nor hard physical activity had a 57% (OR 1.57, 1.07–2.31) increased risk of all-cause death. Both hard (OR 2.77, 2.35–3.26) and light (OR 1.52, 1.32–1.76) physical activity were positively associated with self-reported health. The effect was age dependent. Vigorous physical activity was most beneficial for individuals younger than 40 years old, while moderate physical activity levels prolonged the period in which good self-reported health was likely.

**Conclusions:**

Poor self-reported health and being sedentary were independently associated with an increased risk of mortality in the participants. Furthermore, physical activity prolonged the period of good self-reported health among older adults in two ways: physical activity habits from early adulthood and onwards were beneficial to self-reported health at an advanced age, and self-reported health was dependent on engagement in moderate intensity physical activity after approximately 65 years of age.

## Background

The World Health Organization defines *healthy ageing* as the process of developing and maintaining the functional ability that enables wellbeing in older age. In practical terms, this means creating the environment and opportunities that enable people to be and do what they value [1] to increase wellbeing, participation, and recovery from illness more quickly. This involves, among other things, the option to live in the residence of choice as one ages, called *ageing in place*. Ageing in place is not only perceived as qualitatively better for older people despite illness and disability, but there is also evidence that there may be a socioeconomic benefit to postponing residency in care facilities [2].

Both ideals, *healthy ageing* and *ageing in place*, have gained considerable traction in recent years in Western countries [1, 3], and the scientific community is engaged in investigating factors that will allow for healthy ageing. The most consistent finding in the field of physical activity research in epidemiology is that there is a decline in physical activity with age [4, 5]. Attempting to counteract this trend, both ideals, *healthy ageing* and *ageing in place*, involve strategies for increasing physical activity when implemented [1, 6]. Some strategies for increasing physical activity have been shown to have stronger effects than others; motivational factors, including social support, environmental factors, and experiencing enjoyment from being physically active, are identified as being effective [7].

Physical activity is associated with many health-related outcomes. For older adults, physical activity has been found to be related to cognitive performance [8], frailty [9], weight control, and diabetes [10, 11]. Additionally, physical inactivity among older adults to be associated with a higher risk for cardiovascular disease (CVD) mortality and cancer mortality [12]. Moreover, leisure time physical activity was shown to be inversely associated with all-cause mortality internationally [13, 14] and nationally for ageing adults in a study conducted in Finnmark, Norway [15]. There is also an association between physical activity and mental health among older adults [16]. Despite these beneficial effects, older people tend to be sedentary [17].

The recommendation is for older adults to perform at least 150 min of moderate intensity aerobic physical activity throughout the week [18]. Globally, the majority of older adults fail to meet this recommendation [5, 19]. This is also true for older adults locally in Norway [20, 21]. Women tend to be less physically active than men, especially with regard to leisure-time physical activity [5]. There has been little change in this tendency over the years [22]. There are, however, variations in trends between regions, income groups, and countries [22].

Self-reported health is a general assessment of one’s own health that is strongly associated with a broad range of objective health outcomes, including subclinical and clinical disease [23], health service use [24], and mortality [24–27]. Like physical activity, self-reported health also declines with ageing [24]. Previous studies have found a strong association between physical activity and self-reported health [20, 28, 29]. Nevertheless, there is little investigation regarding the longitudinal relationship between self-reported health and physical activity in older people [30].

As physical activity seems to benefit a broad range of health factors [31] and has a dose-response relationship with mortality [14], it is important to investigate different intensity levels and their potential health benefits among older adults. Previous studies on this have been limited by cross-sectional designs [32–34], the inclusion of only older adults [35], the inclusion of only one sex [36, 37], short follow-up periods [33] or small samples [34, 38]. Knowledge about the long-term effects of physical activity is therefore limited.

The design of and comprehensive data collection in the Tromsø Study (TS) makes it possible to examine the relationship between physical activity and self-reported health throughout life. The TS includes data on the impact of a broad range of other health-related factors, such as comorbidities, mental health symptoms and CVD risk factors, in a large general population sample of both sexes with a broad age range and up to 14 years of follow-up. The objective of this study is to examine how levels of leisure time physical activity are associated with self-reported health trajectories throughout the lifespan via a random coefficient model analysis using repeated measurements at the individual level and to study the effects of self-reported health and physical activity on mortality via a survival analysis.

## Methods

The Tromsø Study (TS) was initiated in 1974 in an attempt to help combat the high mortality due to CVD in Norway. The TS is an ongoing population-based cohort study conducted in Tromsø,the largest city in Northern Norway. Tromsø is situated ∼400 km north of the Arctic Circle and has approximately 67,000 inhabitants. Norway is a high-income country with a high level of education. At baseline in 1994, 64.4% of the subjects had more than 10 years of education (29.8% of them had a college degree). In 2007, this had increased to 70.5%, with 37.4% of the subjects possessing a college degree.

The TS is the most extensive population-based study in Norway. The study consists of seven repeated health surveys, Tromsø 1 (1974), Tromsø 2 (1979–80), Tromsø 3 (1986–87), Tromsø 4 (1994–95), Tromsø 5 (2001), Tromsø 6 (2007–08) and Tromsø 7 (2015–16), which include entire birth cohorts and random population samples (response rates 65–79%) [39].

The original intention of the TS was to investigate the cause of the high mortality rates due to CVD and to develop methods to prevent infarction and stroke. The study has since been expanded to examine a wide range of diseases as well as lifestyle aspects, medication use, sleeping patterns, mental health issues, socioeconomic status and health care utilisation.

### Design

This current study comprises repeated measurements using comprehensive questionnaires, biological samples and clinical examinations. The six surveys of the TS had the same general design. A questionnaire was enclosed in the invitation to all surveys, including questions about a wide range of diseases and symptoms, lifestyle aspects, use of medication, socio-economic status, and use of health-care services. Blood samples and measurements of blood pressure, height, and weight were collected during the physical examination. All participants are being followed up with regard to mortality and disease incidence [39]. Tromsø 4 is the largest survey to date and includes all age groups (range 25–97 years); thus, Tromsø 4 was chosen as the baseline for our study.

Longitudinal studies are used to investigate age-related developmental changes. The survival analysis utilized a single cohort design. The individuals were grouped according to physical activity level at the study’s baseline and followed over time. An accelerated longitudinal design includes multiple single cohorts, each starting at a different age. The principal benfit of an accelerated longitudinal design is its capacity to stretch the age range of interest over a shorter period than would be possible with a single-cohort longitudinal design [40]. This study utilised a mixed effect model to describe self-reported health across ages, with random effects for individual and time [41]. Participants aged 25–87 years who had at least two measurements each were included.

The continuous overlap of individual trajectories allowed us to stretch the age range of interest based on an average follow-up time of 14 years. A disadvantage was missing data, which can be a problem when there is an age cohort effect [40]. Consequently, this study used inverse probability weighting (IPW), a common method for correcting such bias [42].

The cohort was observed for up to 21.3 person years, and the average follow-up time was 18.8 years. During this period, 5508 subjects died, giving an overall incidence rate of .018 deaths per person year. The Norwegian mortality incidence rate improved from 0.011 for men and 0.0093 for women in 1985 to 0.0078 for men and 0.0084 for women in 2015 [43]. In Western Europe, the mortality incidence rate has increased from 0.010 to 0.0096 in the same period. The study population has, therefore, a higher mortality rate than the national average, but the rate has improved over the observation period more quickly, mainly due to improvements in cardiovascular risk factors [44]. The three major causes of death in Norway were CVD (245 per 100,000 people), neoplasms (221 per 100,000 people) and neurological disorders (101 per 100,000 people). The major risk factors are behavioural (i.e., dietary, tobacco and low physical activity) and metabolic (i.e., high blood pressure, cholesterol and high BMI). The proportion of inactive subjects increases with increasing age, especially among females [21], and the supplementary material (Additional Files 1 and 2) shows the physical activity levels for females and males according to age at baseline. The proportion of inactive females was 57.6% (all ages) versus 49.7% of men at baseline. During the follow-up period, a lower proportion of inactive subjects were registered, especially among women. In 2007, 20.3% of the females were inactive, compared with 19.7% of the men.

### Sample

**Fig. 1:**
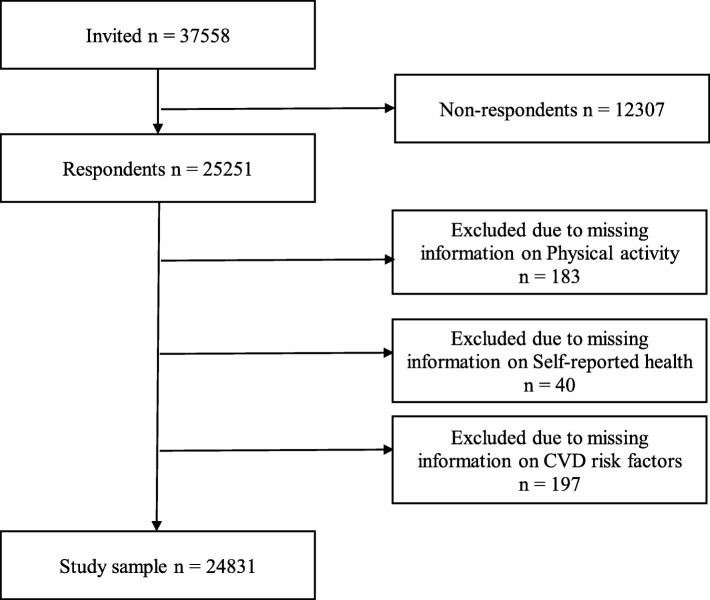
Flow chart of the study sample

A total of 25,251 women and men participated in Tromsø 4 [33]. For the survival analysis, the study followed the participants from study entry in 1994 to the day of death or the end of follow-up on December 31, 2015, whichever came first. Subjects who had missing values for physical activity (*n* = 183), self-reported health (*n* = 40), or CVD risk factors (*n* = 197) were excluded. In total, there were 24,831 participants (52% women) aged 25–97 years in the baseline analysis. Subjects who participated again in Tromsø 5 (*n* = 6093) and Tromsø 6 (*n* = 10,534) had their self-reported health and risk factor values updated at the time of examination. Figure 1 shows the flow chart of the study sample.

**Fig. 2:**
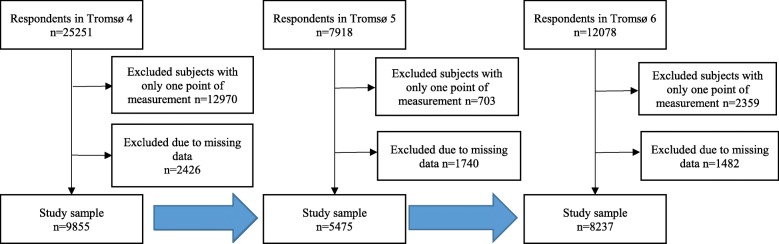
Flow chart of inclusion in the random coefficient analysis

For the random coefficient analysis, the participants were followed from the day of study entry in 1994 to the day of study exit in 2007–8. Random coefficient analysis requires two or more measurement points. In total, 25,251 women and men from the 1994 cohort who participated in the Tromsø 4 survey were included, and participants with missing data on self-reported health, physical activity, mental health or CVD risk factors were excluded. Thus, the analysis included 9855 participants (52% women) aged 25–80 years at the first examination. Of these individuals, 5475 (56%) were re-examined in Tromsø 5, and 8237 (84%) were re-examined in Tromsø 6. Figure 2 shows the inclusion for the random coefficient analysis.

### Variables

The outcomes of interest were self-reported health and all-cause mortality. Self-reported health was based on the question “What is your current state of health?”, and the response options were “Poor”, “Not so good”, “Good”, and “Very good”. The time and cause of death were retrieved from the Norwegian National Causes of Death Registry. Norway has a personal identification system that permits exact matching of population register data, and the degree of coverage in the registry is nearly complete [45].

Leisure time physical activity levels and age were the independent variables of interest, and the other variables were treated as confounders. Physical activity was defined in Tromsø 4 and 5 by two questionnaire items that asked about weekly average hours and level of physical activity: a) Hard physical activity was defined as an activity level that involved sweating/loss of breath. No activity was categorized as “sedentary”, less than 1 h of activity was scored as “some high-intensity activity”, 1–2 h was considered “moderate high-intensity activity” and 3 or more hours was considered “vigorous high-intensity activity”. b) Light physical activity was self-reported activity that did not involve sweating/loss of breath and was categorized as follows: none, less than 1 h, 1–2 h or 3 or more hours. The results were based on responses to questions that the participant answered regarding the weekly average of each physical activity level over the past year. In Tromsø 6, the validated Saltin and Grimby scale [46] was used: “Exercise and physical exertion in leisure time”: “Reading, watching TV or other sedentary activity” (sedentary); “Walking, cycling or other forms of exercise at least 4 hours per week” (low); “Participation in recreational sports, heavy gardening, etc. at least 4 hours a week” (moderate); and “Participation in hard training or sports competitions regularly several times a week” (vigorous).

Comorbid diseases were self-reported specific medical conditions. The severity of a disease affects the level of self-reported health. Therefore, this study utilised the previously developed Health Impact Index (HII) [47] to measure comorbid conditions. The HII classifies individuals with comorbid diseases according to the impact that each condition has on self-reported health by assigning a weight to each condition. The HII score is equal to the subject’s total score for all conditions; thus, the HII considers joint effects and the severity of comorbid conditions. Mental health symptom scores were based on two validated self-reported instruments: the CONOR Mental Health Index (CONOR-MHI), which was used in Tromsø 4, and the Hopkins Symptoms Checklist 10 (HSCL-10), which was used in Tromsø 5 and Tromsø 6. These two instruments have previously shown good correlations (r = .9) in a validation study [40], and the variables were standardized for better longitudinal comparison in the regression model.

Education level and daily smoking were collected via questionnaires. Specially trained personnel collected measurements of body weight and height, blood pressure, resting heart rate, and non-fasting blood samples using standard methods. We calculated body mass index (BMI) as the weight in kilograms divided by the square of the height in metres and was grouped according to the World Health Organization (WHO) (2018) BMI classification for underweight, normal, overweight and obese (< 18.5 kg/m^2^, 18.5–24.9 kg/m^2^, 25–29.9 kg/m^2^ and ≥ 30 kg/m^2^, respectively). Blood samples were analysed for total cholesterol using standard methods at the Department of Laboratory Medicine at the University Hospital of North Norway.

### Statistical analysis

**Fig. 3:**
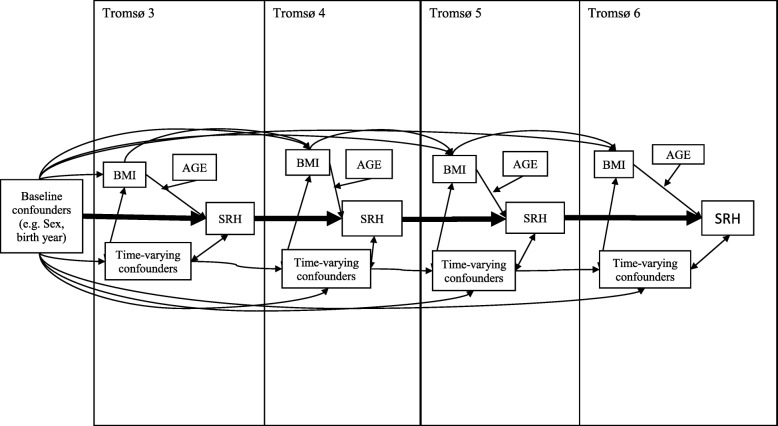
Directed acyclic graph showing the conceptual model and its translation into a statistical model

Figure 3 shows the conceptual model and how it translates into a statistical model. Table 1 describes sample characterisation versus period, including the common Pearson’s chi-squared test for the categorical variables and one-way ANOVA for the continuous variables. Table 2 compares self-reported health by age versus physical activity level. The analytical goal was to describe covariation among age, physical activity and self-reported health. Because self-reported health is a categorical variable with ordered categories, a random coefficient proportional odds model was used to assess how self-reported health changed over time [41]. The units used for a longitudinal context with repeated measurements are occasions (j), and the clusters are subjects (i). Time is represented as cohort_i,_ age_ij_, and period. Cohort_i_ is the birth year. Age_ij_ is the age on the date of the survey for each subject. Period_i_ is the participation year. We describe time by the equation age_ij_ = period_i_ - cohort_j_. One time variable was thus collinear with the two others and would be left out of the model; therefore, we could consider a model that included the two time scales (age_ij_ and period_i_) as covariates as well as PA, sex, physical examination measurements (BMI, systolic blood pressure, resting heart rate and total cholesterol), pathology (comorbidity and mental health symptoms), education and smoking. The interaction between physical activity and age was also modelled.

**Table 1:** Sample characteristics over three surveys with age and gender adjusted mean with 95% confidence intervals. The Tromsø Study 1994–2008

	Tromsø4 1994–1995					Tromsø5 2001–2002					Tromsø6 2007–2008					*P*-value
	Freq.	Percent	Adjusted rate	95% CI		Freq.	Percent	Adjusted rate	95% CI		Freq.	Percent	Adjusted rate	95% CI		
**Self-reported health**																
*Poor*	*598*	*0.024*	*0.029*	*0.026*	*0.031*	*120*	*0.020*	*0.014*	*0.010*	*0.018*	*540*	*0.051*	*0.044*	*0.040*	*0.048*	*< 0.001*
*Not so good*	*6894*	*0.278*	*0.318*	*0.312*	*0.324*	*1982*	*0.325*	*0.269*	*0.252*	*0.287*	*2989*	*0.284*	*0.249*	*0.240*	*0.259*	
*Good*	*13,750*	*0.554*	*0.533*	*0.527*	*0.540*	*3346*	*0.549*	*0.525*	*0.504*	*0.547*	*5443*	*0.517*	*0.495*	*0.484*	*0.506*	
*Very good*	*3589*	*0.145*	*0.120*	*0.116*	*0.124*	*645*	*0.106*	*0.110*	*0.094*	*0.126*	*1562*	*0.148*	*0.151*	*0.143*	*0.160*	
**Light physical activity**																
*None*	*3018*	*0.123*	*0.133*	*0.129*	*0.138*	*464*	*0.081*	*0.058*	*0.052*	*0.065*	*50*	*0.012*	*0.009*	*0.006*	*0.012*	*< 0.001*
*< 1 h/week*	*3823*	*0.154*	*0.148*	*0.144*	*0.153*	*672*	*0.117*	*0.126*	*0.109*	*0.143*	*1260*	*0.292*	*0.322*	*0.302*	*0.342*	
*1-2 h/week*	*8365*	*0.337*	*0.327*	*0.321*	*0.333*	*1944*	*0.334*	*0.302*	*0.283*	*0.322*	*998*	*0.233*	*0.219*	*0.200*	*0.237*	
*≥ 3 h/week*	*9543*	*0.385*	*0.391*	*0.385*	*0.398*	*2745*	*0.468*	*0.436*	*0.415*	*0.458*	*1951*	*0.462*	*0.372*	*0.356*	*0.389*	
**Hard physical activity**																
*Sedentary*	*12,459*	*0.502*	*0.549*	*0.543*	*0.555*	*2257*	*0.370*	*0.299*	*0.281*	*0.317*	*2129*	*0.202*	*0.193*	*0.184*	*0.202*	*< 0,001*
*Some high intensity*	*4987*	*0.201*	*0.183*	*0.178*	*0.188*	*1674*	*0.275*	*0.277*	*0.256*	*0.298*	*6340*	*0.602*	*0.540*	*0.529*	*0.551*	
*Moderate high intensity*	*4885*	*0.197*	*0.177*	*0.173*	*0.182*	*1488*	*0.244*	*0.246*	*0.225*	*0.267*	*1893*	*0.180*	*0.185*	*0.176*	*0.194*	
*Vigorous high intensity*	*2500*	*0.101*	*0.091*	*0.087*	*0.094*	*674*	*0.111*	*0.096*	*0.084*	*0.108*	*172*	*0.016*	*0.021*	*0.017*	*0.025*	
**Smoking**																
*Daily smokers*	*9019*	*0.363*	*0.357*	*0.351*	*0.363*	*1714*	*0.274*	*0.286*	*0.264*	*0.309*	*2076*	*0.190*	*0.154*	*0.145*	*0.163*	*< 0.001*
	*Count*	*Mean*	*Adjusted rate*	*95% CI*		*Count*	*Mean*	*Adjusted rate*	*95% CI*		*Count*	*Mean*	*Adjusted rate*	*95% CI*		*P-value*
Comorbidity	*24,831*	*0.950*	*1.115*	*1.091*	*1.138*	*7004*	*1.437*	*1.192*	*1.138*	*1.246*	*9488*	*1.401*	*1.283*	*1.247*	*1.319*	*< 0.001*
BMI	*24,831*	*25.227*	*25.434*	*25.382*	*25.485*	*7004*	*26.666*	*26.354*	*26.198*	*26.510*	*9488*	*26.929*	*26.716*	*26.617*	*26.816*	*< 0.001*
RHR	*24,831*	*72.467*	*72.723*	*72.556*	*72.890*	*7004*	*70.085*	*70.055*	*69.512*	*70.597*	*9488*	*64.957*	*64.760*	*64.523*	*64.996*	*< 0.001*
SBP	*24,831*	*135.144*	*138.255*	*137.998*	*138.511*	*7004*	*137.007*	*133.643*	*132.957*	*134.329*	*9488*	*135.451*	*132.603*	*132.219*	*132.987*	*< 0.001*
Total cholesterol	*24,831*	*6.088*	*6.267*	*6.251*	*6.283*	*7004*	*6.099*	*6.032*	*5.993*	*6.071*	*9488*	*5.612*	*5.505*	*5.482*	*5.528*	*< 0.001*

*SRH* self-reported health, *PA* physical activity, *BMI* body mass index, *RHR* resting heart rate, *SBP* systolic blood pressure, *CI* 95% confidence intervals, *Adj.rate* age and gender standarized rates

*P*-value is based on chi square. SRH: Pearson chi2(6) = 324.5484, p < 0.001; Hard physical activity: Pearson chi2(6) = 6.3e+ 03, p < 0.001; Light physical activity Pearson chi2(6) = 1.2e+ 03, p < 0.001; Daily smokers Pearson chi2(2) = 981.7926, *p* < 0.001. *P*-value for the continuous variables are based upon ANOVA grouped on survey time: Comorbidity: F(2) = 385.46, *p* < 0.001; BMI: F (2) = 699.31, *p* < 0.001; RHR: F (2) = 1481.70, *p* < 0.001; SBP: F (2) = 26.44, *p* < 0.001; Tot.cholesterol F (2) = 612.58, *p* < 0.001

**Table 2:** Self-reported health levels by 10-year age groups and various measures of physical activity levels over three surveys. The Tromsø Study 1994–2008

Hard physical activity		Sedentary		Some high intensity		Moderate high intensity		Vigorous high intensity			
	Age group	n	SRH	n	SRH	n	SRH	*n*	SRH	*N*	*p*-value
Tromsø 4	25–29	734	79.6%	615	87.1%	677	92.5%	444	92.3%	2470	< 0.001
	30–39	2052	79.5%	1526	84.2%	1546	88.6%	712	89.9%	5836	
	40–49	2941	69.0%	1513	78.8%	1462	83.7%	689	85.2%	6605	
	50–59	2433	54.6%	817	69.6%	737	75.5%	390	74.9%	4377	
	60–69	2144	43.3%	373	60.3%	345	64.2%	192	62.5%	3054	
	70–79	1733	38.5%	145	59.3%	140	60.0%	93	65.6%	2111	
	≥80	559	32.9%	27	57.7%	14	28.6%	3	100.0%	603	
Tromsø 5	30–39	112	81.1%	176	86.9%	161	88.2%	82	86.6%	531	< 0.001
	40–49	305	73.3%	381	82.1%	381	85.5%	179	85.4%	1246	
	50–59	317	51.8%	275	67.5%	214	68.7%	93	73.9%	899	
	60–69	737	52.4%	579	66.1%	487	67.4%	219	77.4%	2022	
	70–79	764	42.5%	276	57.2%	249	65.4%	106	60.2%	1395	
	≥80	109	40.6%	33	51.6%	22	54.5%	11	66.7%	175	
Tromsø 6	30–39	90	62.2%	216	75.5%	113	91.2%	24	100.0%	443	< 0.001
	40–49	540	58.1%	1414	76.1%	629	87.5%	79	97.5%	2662	
	50–59	454	53.6%	1470	69.2%	372	81.6%	33	93.9%	2329	
	60–69	678	49.8%	2392	62.5%	629	79.6%	35	91.4%	3734	
	70–79	354	36.0%	920	57.5%	194	73.4%	4	100.0%	1472	
	≥80	159	30.3%	204	51.7%	34	50.0%	0		397	
Light physical activity		None		< 1 h/week		1-2 h/week		≥3 h/week			
	Age group	n	SRH	n	SRH	n	SRH	n	SRH	N	*p*-value
Tromsø 4	25–29	218	79.6%	371	82.5%	826	86.7%	1057	91.0%	2472	< 0.001
	30–39	492	79.7%	946	82.0%	2086	84.2%	2306	86.4%	5830	
	40–49	646	69.8%	1195	72.9%	2488	76.3%	2284	79.3%	6613	
	50–59	551	50.4%	656	59.2%	1520	63.7%	1670	66.9%	4397	
	60–69	439	32.6%	348	41.1%	920	49.6%	1382	55.6%	3089	
	70–79	501	23.4%	272	26.6%	520	44.4%	842	58.0%	2135	
	≥80	242	21.9%	91	35.2%	123	36.6%	149	51.4%	605	
Tromsø 5	30–39	22	86.4%	60	83.1%	172	87.2%	282	86.9%	536	< 0.001
	40–49	53	73.6%	179	77.0%	461	79.9%	573	82.2%	1266	
	50–59	63	59.0%	119	49.6%	368	62.2%	464	67.2%	1014	
	60–69	175	39.4%	250	53.7%	792	58.0%	1228	65.8%	2445	
	70–79	228	33.5%	199	36.3%	526	51.1%	720	59.1%	1673	
	≥80	45	27.9%	31	22.6%	58	51.8%	74	63.4%	208	
Tromsø 6	30–39	0		51	54.9%	25	68.0%	27	59.3%	103	< 0.001
	40–49	7	42.9%	312	60.6%	191	68.1%	295	71.5%	805	
	50–59	7	42.9%	304	53.2%	224	68.5%	380	62.0%	915	
	60–69	23	69.6%	490	52.9%	417	58.7%	864	58.5%	1794	
	70–79	15	26.7%	191	47.1%	205	51.5%	499	56.6%	910	
	≥80	6	16.7%	44	34.1%	49	35.4%	133	56.8%	232	

Note: SRH is dicotomised between poor/not so good versus good/very good. Percent in the table displays those who are at good or very good Health. A chi-square test indicates that there was a significant difference in the different SRH levels and exercise level in all surveys

Tromsø 4 hard PA: LR chi2(9) = 3503.33

Tromsø 5 hard PA: LR chi2(8) = 582.58

Tromsø 6 hard PA: LR chi2(8) = 856.39

Tromsø 4 light physical activity: LR chi2(9) = 3261.85

Tromsø 4 light physical activity: LR chi2(8) = 593.20

Tromsø 4 light physical activity: LR chi2(8) = 77.69

The TS has had high overall response rates. However, only subjects who had participated in a minimum of two surveys were included in the analyses of the current study, which introduced the risk of selection bias. Loss to follow-up always causes a loss of information that cannot be recovered. The concern is that loss to follow-up can be due to known or underlying conditions (e.g., there was an observed decline because some participants were ill at baseline and thus had low physical activity levels). The most straightforward approach to dealing with missing data is to restrict the analysis to complete cases (CCs), i.e., individuals with no missing values. A total of 12,241 of the 28,409 subjects were used in the analysis, which could induce bias. The use of the IPW method did not significantly change the model [42]. No variables of interest became significant that were non-significant in the CC model, or vice versa. Hence, the IPW model based on the probability of follow-up did not change the main findings but, as expected, reinforced the observed decline. The population average is therefore most likely slightly lower than that of the CC model. Therefore, the IPW model is the main model reported in this study.

Furthermore, Cox proportional hazard regression models were used to estimate hazard ratios (HRs) for all-cause mortality related to physical activity and self-reported health using baseline values for age and sex in addition to comorbidities, mental health symptoms and CVD risk factors as time-dependent covariates updated in Tromsø 5 and Tromsø 6. The time at risk was person-time, measured in days from the first participation date. The proportional hazard assumption was verified for physical activity by visual inspection of log-log survival curves and by tests of Schoenfeld residuals. Self-reported health was added as a time-dependent variable to include its interaction with time. Statistical analysis was performed using STATA 14 [48].

## Results

### Sample characteristics

The sample included *n* = 24,831 subjects. The mean age of the participants increased between the surveys, so all estimates were standardized for age and sex. The mean age was 48.1 ± 14.8 years in Tromsø 4, 62.8 ± 11.4 years in Tromsø 5 and 61.3 ± 11.1 years in Tromsø 6. Table 1 shows the age- and sex-adjusted overview of the sample characteristics across the three study surveys. Seventy percent of the subjects reported good or very good health in Tromsø 4; the adjusted rate (65%) (*n* = 24,831) decreased to 63% in Tromsø 5 (*n* = 6093) and was 64% in Tromsø 6 (*n* = 10,534). Health-related behaviours changed during the same period: the number of physically active subjects increased significantly, and daily smoking nearly halved. When comparing men and women, we see that physical activities benefit all (Additional File 3). Comorbidity increased throughout the study period. The sex- and age-adjusted estimates for blood pressure and cholesterol declined, while BMI increased (Table 1).

### Self-reported health and physical activity according to age

Table 2 shows the association between physical activity and self-reported health by age group (*n* = 24,831 in Tromsø 4, *n* = 6093 in Tromsø 5 and *n* = 10,534 in Tromsø 6). Both were inversely associated with age, and self-reported health was positively associated with physical activity in every age group. Subjects who reported no physical activity also reported the lowest levels of self-reported health, and the decreases with age were more substantial for this group than for subjects who reported physical activity. Compared with light physical activity, vigorous physical activity was more strongly associated with self-reported health. A chi-square test indicated that there was a significant difference in the different self-reported levels of health and exercise levels in all surveys (Table 2).

Table 3 shows the estimates for the associations between light and vigorous physical activity and self-reported health adjusted for age, sex, comorbidity, education, smoking and CVD risk factors (*n* = 9855 at baseline). The estimates from the IPW model were weighted to account for those lost to follow-up. Self-reported hard physical activity was nearly three times (OR 2.77, 95% CI: 2.35, 3.26) more beneficial to self-reported health than no hard physical activity. Self-reported light physical activity had a 52% (OR 1.52, 95% CI 1.32, 1.76) greater positive effect on self-reported health compared to no light physical activity (Table 3).

**Table 3:** Results from the random-coefficient proportional odds model with estimates for the association of subject-specific factors on Self-Reported Health

	Basic model			Complete case model			IPW (missing)			
	OR	(95% CI)		OR	(95% CI)		OR	(95% CI)		
**Hard physical activity**										
Sedentary (reference)	1.00			1.00			1.00			
Some high intensity	1.25	(1.14,	1.37)	1.10	(0.76,	1.59)	0.64	(0.35,	1.18)	
Moderate high intensity	1.97	(1.77,	2.19)	2.09	(1.38,	3.15)	1.66	(0.83,	3.31)	
Vigorously high intensity	2.77	(2.35,	3.26)	8.89	(4.89,	16.17)	9.99	(3.48,	28.71)	
**Light physical activity**										
None (reference)	1.00			1.00			1.00			
< 1 Hour	1.09	(0.93,	1.28)	0.67	(0.36,	1.24)	0.60	(0.21,	1.73)	
1–2 h	1.34	(1.16,	1.55)	0.59	(0.33,	1.04)	0.54	(0.20,	1.44)	
> 3 h	1.52	(1.32,	1.76)	0.47	(0.26,	0.83)	0.39	(0.14,	1.04)	
**Interactions**										
hardPA#c.age										
Inactive (reference)				1.00			1.00			
Some high intensity				1.02	(0.96,	1.09)	1.13	(1.02,	1.26)	
Moderate high intensity				0.99	(0.92,	1.06)	1.05	(0.93,	1.18)	
Vigorously high intensity				0.79	(0.71,	0.89)	0.79	(0.65,	0.95)	
lightPA#c.age										
Inactive (reference)				1.00			1.00			
< 1 Hour				1.09	(0.97,	1.21)	1.10	(0.91,	1.31)	
1–2 h				1.15	(1.05,	1.28)	1.17	(0.99,	1.38)	
> 3 h				1.23	(1.11,	1.35)	1.28	(1.08,	1.51)	
**Time varying confounders**										
Body mass index										
< 18.49 kg/m2	0.53	(0.33,	0.85)	0.58	(0.39,	0.87)	0.83	(0.38,	1.78)	
18.5–24.99 Kg/m2	1.00			1.00			1.00			
25–29.99 kg/m2	0.71	(0.65,	0.78)	0.73	(0.68,	0.80)	0.64	(0.56,	0.74)	
> 30 kg/m2	0.40	(0.36,	0.46)	0.44	(0.39,	0.49)	0.32	(0.26,	0.39)	
Comorbidity (HII)	0.74	(0.72,	0.76)	0.74	(0.73,	0.76)	0.73	(0.70,	0.76)	
Mental distress (std)	0.37	(0.35,	0.39)	0.38	(0.36,	0.40)	0.34	(0.32,	0.37)	
Resting heart rate (std)	0.86	(0.83,	0.90)	0.87	(0.84,	0.91)	0.81	(0.76,	0.87)	/cut1
Systolic Blood Pressure (std)	1.10	(1.06,	1.15)	1.08	(1.04,	1.12)	1.19	(1.12,	1.28)	/cut2
Total cholesterol (std)	1.04	(1.00,	1.09)	1.04	(1.00,	1.08)	1.07	(1.01,	1.14)	/cut3
Daily smoker	0.76	(0.69,	0.83)	0.76	(0.69,	0.82)	0.77	(0.67,	0.89)	
**Education level**										
Primary school (reference)	1.00			1.00			1.00			
tech.school, middle school, vocatitional school, or high school diploma (3–4 years)	1.39	(1.25,	1.54)	1.35	(1.23,	1.49)	1.54	(1.33,	1.77)	
College/university	2.76	(2.45,	3.11)	2.54	(2.28,	2.84)	4.15	(3.51,	4.91)	
/cut1: Poor	−9.17	-(9.53,	−8.81)	−9.21	-(9.79,	−8.64)	−13.90	-(14.91,	−12.88)	
/cut2: Not so good	−4.39	-(4.69,	−4.10)	−4.76	-(5.31,	−4.22)	−7.87	-(8.82,	−6.92)	
/cut3: Good	0.48	(0.21,	0.75)	−0.25	-(0.79,	0.28)	−1.33	-(2.25,	−0.41)	
**Random part of the model**										
var. (constant)	3.15	(2.90,	3.42)	2.24	(2.05,	2.44)	8.37	(7.77,	9.01)	

Basic:Wald chi2(19) = 3766.27, *p* < 0.0001

CC model: Wald chi2(25) = 4564.77 Prob > = chibar2 = 0.0000

IPW (Missing): Wald chi2(25) = 2331.80, p < 0.0001

*OR* odds ratio, *CI* Confidence interval, *PA* physical activity, *CVD* Cardiovascular disease, *std.* standardised

**Fig. 4:**
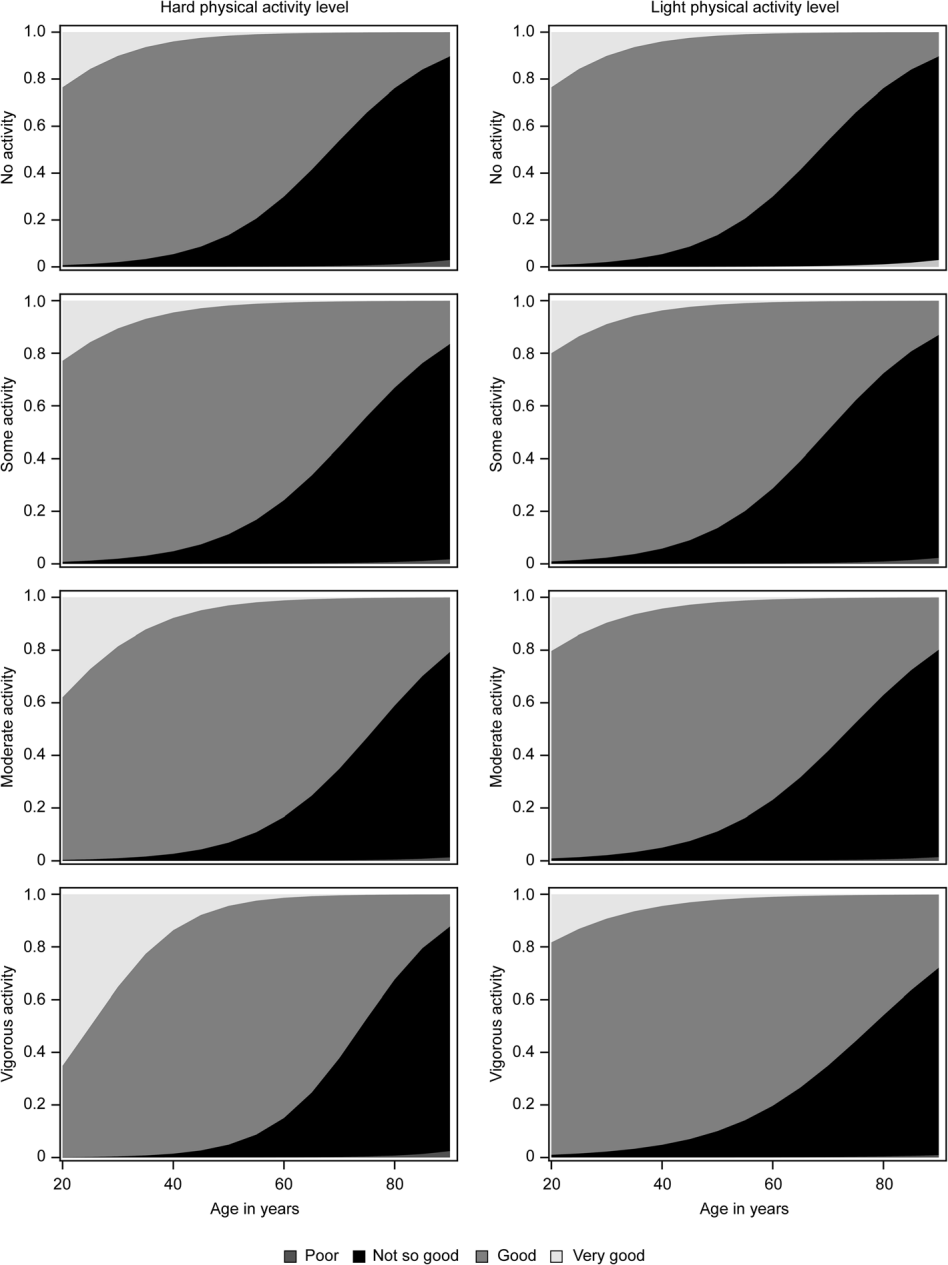
Self-reported health levels according to age and activity level. The vertical axis shows the category probability, and the horizontal axis shows the age. The graph shows that vigorous physical activity affects the probability of very good self-reported health, especially at ages below 40 years (left column) and that light physical activity prolongs the period for which good self-reported health is likely (right column)

The inclusion of the interaction terms in the main model showed that the positive effect of physical activity depended on age. Figure 4 presents the category probabilities according to age and physical activity levels. It is based on the main model in Table 3 and shows the estimated category distribution for self-reported health at a given physical activity level for the age range from 20 to 90 years. The overall interpretation is that the areas representing the most beneficial self-reported health categories (i.e. good and very good) increase with increasing physical activity levels. We see the same trend for light physical activity, but here the positive effect is most pronounced at ages above 65 years. More specifically, we see how hard physical activity affects the probability of having very good self-reported health, especially among subjects younger than 40 years; for those older than 40, light physical activity lengthened the period during which good self-reported health was likely. For example, 25-year-old subjects who reported engaging in vigorous physical activity had a 99.8% probability of good self-reported health, an effect that decreased by 24% (OR 0.76, 95% CI 0.62, 0.94) with every 10-year increase in age. At age 25, those who reported vigorous hard physical activity had a 47.2% probability of having very good self-reported health.

To further control for bias due to prior exposure (e.g., people who are already sick will have lower physical activity levels), IPW was used based on the propensity score for each physical activity level. IPW did not change the main results of the model, which means that no factors that were significant became non-significant or vice versa. Visual inspection of the figure also showed that IPW did not change the main relationship between the curves. The figure also shows that the difference in self-reported health at 25 years was attenuated (i.e., 99.9% of the vigorous physical activity group and 99.1% of the group with no physical activity had good self-reported health). However, the drop in self-reported health was steeper for all activity groups. Compared to the results of the CC analysis, vigorous high-intensity activity levels passed the 50% probability level approximately 6 years earlier (71.5 years), and participants who described themselves as sedentary passed the same level approximately 4 years earlier (66.5 years).

Light physical activity did not affect very good levels of self-reported health as much as hard physical activity did, but it still postponed the likelihood of not having good self-reported health by ~ 10–12 years in the fully fitted model. Subjects who reported engaging in light physical activity > 3 h per week at baseline had an 11.3% probability of reporting very good self-reported health at age 25, which was lower than the probability for those who engaged in hard physical activity; nonetheless, they still had a 98.9% probability of good self-reported health at age 25, an effect that decreased by 23% (OR 1.23, 95% CI: 1.02, 1.49) with every 10-year increase in age.

**Fig. 5:**
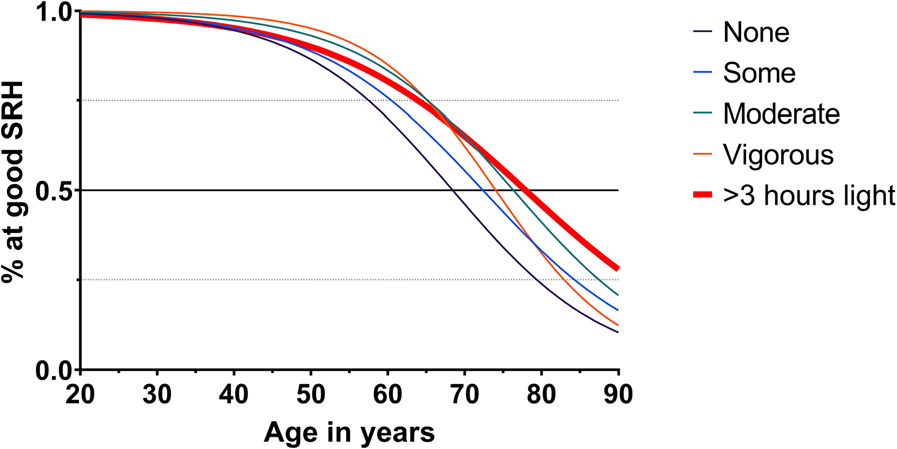
Probability of being in good self-reported health according to age and activity levels in the fully fitted model. The vertical axis shows the category probability, and the horizontal axis shows the age. The model was controlled for all confounders and weighted to adjust for missing data

Ageing alone was associated with a decline in self-reported health throughout life (OR 0.34, 95% CI: 0.28, 0.40). However, the model predicted that both light and hard physical activity would prolong the period during which a person was likely to report good self-reported health. Fig. 5 presents the response (self-reported health, *SRH*_*ij*_) as a function of age and physical activity levels; this response is presented as category probabilities of a self-reported health score of good or very good. When we compared the probability of responding “Good” versus “Not so good” for the different physical activity levels, we observed that those who reported vigorous high intensity activity levels had only an 11.9% probability of good self-reported health at age 90, while for those who reported engaging in moderate high-intensity physical activity, the probability was 20.8%. For those engaging in light physical activity (> 3 h), the probability of having good self-reported health at age 90 was 27.2%, which was the highest probability for that age. The 50% line indicates the cut-off level for the probability of reporting good self-reported health, i.e., light physical activity > 3 h per week was associated with good self-reported health up to age 75 years, which was 8 years longer than among those who reported no light physical activity. Furthermore, moderate physical activity was associated with good self-reported health up to 74 years of age. It was also possible to observe how the lines crossed. Moderate high-intensity physical activity was thus most beneficial from approximately 63 years of age, while light physical activity (> 3 h) was more beneficial starting at approximately 70 years of age according to the fully fitted model. Those who reported no light or hard physical activity had less than a 50% probability of self-reporting good health at age 65 years and older.

### The effect of self-reported health and physical activity on mortality

Subjects (*n* = 24,831) who reported engaging in neither light nor hard physical activity had a 43% increased risk of all-cause death (OR 1.57, 95% CI 1.07, 2.31). Subjects who did not report vigorous physical activity could still report light physical activity levels, and vice versa. Consequently, subjects who reported no hard physical activity had a 32% increased risk of all-cause death, and subjects who reported no light physical activity had a 23% increased risk after adjustment for comorbidity, mental health symptoms, CVD risk factors, smoking and education (Table 4). Positive effects on mortality were observed for both light and hard physical activity. There was also a combined effect of light and hard physical activity (see Supplementary Table 3). When estimating the effects of different combinations of light and hard physical activity, light physical activity had a cumulative effect on both self-reported health and mortality. Light physical activity was especially effective for those who did not report any hard activity.

## Discussion

We found that physical activity reduced overall mortality. The main finding, however, was that achieving a beneficial level of physical activity for one’s age extended self-reported good health for up to 15 years. As the mean age of the population has increased, public health priorities have promoted the importance of functional ability and quality of life for postponing individuals’ need for home-based care and nursing home services [1]. Physical activity is generally associated with higher levels of self-reported health [29]. However, the vast majority of adults do not meet the guidelines for physical activity [19]. Few studies have examined how levels of leisure time physical activity in early adulthood are associated with self-reported health trajectories throughout life. One study indicated an inverse link between physical activity and self-reported health in a Spanish population and found that the strongest effect of physical activity on self-reported health occurred for people aged 50–69 years [30]. Our results support these findings.

### Physical activity reduced overall mortality

In the current study, we found that being physically active reduced overall mortality. Both light and hard physical activity had beneficial effects on the period during which the subjects reported good self-reported health, and this result remained after adjustment for comorbidities, mental health symptoms, CVD risk factors, smoking and education level. It is consistent with previous reports that an increase in physical activity levels minimises the burden on health and social care by enabling healthy ageing [5]. Previous studies have shown that older age groups report lower physical activity levels than younger age groups and that women report lower physical activity levels than men, especially for leisure time physical activity, when measured by both subjective and objective methods [5]. Our study shows that physical activity habits in early adulthood significantly predict physical activity habits in older age and that self-reported health in older age depends on physical activity habits established in early adulthood and onwards. Both light and hard physical activity had beneficial effects on survival, while being sedentary resulted in an increased risk of mortality.

### Physical activity was beneficial for self-reported health trajectories

Physical activity prolonged the period of good self-reported health throughout life. Both light and hard physical activity were positively associated with self-reported health. Both self-reported health and physical activity were inversely related to age, and the main finding of this study was that achieving the appropriate level of physical activity for one’s age extended the duration of good self-reported health for up to 15 years.

Vigorous high-intensity physical activity was most effective for people up to 40 years of age, but after ~ 63 years of age, a moderate high-intensity physical activity level was more beneficial for self-reported health. This positive effect on self-reported health was also improved by weekly light physical activity, such as walking and other light activities. The results of this study show that when people are in their 60s, they are likely to experience poor self-reported health if they have a low physical activity level, and this finding is probably related both to the lack of physical activity because of illness and, conversely, to illness because of a lack of physical activity.

However, the main effect was observed for hard physical activity. The effect of hard physical activity on self-reported health was more than three times as high as the effect of light activities. An interesting finding in the current study was that participants who reported a physical activity level that fell within the WHO recommendations [31] experienced a period of good self-reported health that was extended by 10–20 years. According to the WHO [31], the optimal amount of physical activity is approximately 30 min a day. The current study’s results indicate that a daily walk lasting approximately half an hour is enough to reduce mortality but not enough to achieve improved self-reported health. The beneficial level of physical activity is age dependent and involves a combination of light and hard physical activity. The most favourable trajectory in terms of both mortality and self-reported health seems to involve a moderate physical activity level after approximately 65 years of age.

Previous reports from the TS have described gender differences in physical activity [49], but we found no significant gender differences in mortality or in how physical activity affects self-reported health. This finding is in line with the study by Lera-Lopez et al. [30]. Because both sexes received the same benefit from physical activity in this study, the lower physical activity and self-reported health levels of women at older ages are of concern.

### Limitations and strengths

The main strength is the longitudinal design with repeated surveys with high participation rates conducted within the same community. This allows us to demonstrate how the prevalence of physical activity affects trends in self-reported health and mortality rates, as well as to track the development of risk factors in the same individual up to three times during a period of up to 21 years. A limitation of the current analysis is the effect of possible changes in physical activity levels. The models were cross-sectional time series in which physical activity levels may change between examinations. Comorbid disease (OR 0.96, 95% CI 0.93, 0.98) and mental health symptoms (OR 0.82, 95% CI 0.80, 0.85) also decreased physical activity levels. Although the model controls for these confounders, they could also represent prior exposure (e.g., if physical activity levels were low because the participants were already ill). The most plausible explanation for this finding is that the decreased physical activity levels with age are due to an increased comorbidity burden, which accelerates the decline in self-reported health. Furthermore, there is a possibility that there is a limitation to the accuracy of the data due to self-reporting.

A significant strength of the current study is the study design, which allowed an investigation of the relationship between physical activity and self-reported health throughout the lifespan from adulthood to older age and an examination of the impact of a broad range of covariates in a large general population sample of both women and men with up to 14 years of follow-up. The empirical evidence presented concerns the design of public health-care advice promoting ageing in place, healthy ageing and physical activity. However, it is not possible to make causal conclusions based on a single study. Based on our results, we cannot conclude that starting to exercise at an older age has a beneficial effect on mortality and self-reported health, but physical activity in early life predicts physical activity in later stages of life, which is in line with previous studies [49].

## Conclusion

The main findings of this study were that both self-reported health and physical activity were inversely related to age and that achieving the appropriate level of physical activity for one’s age extended the duration of good self-reported health for up to 15 years. Physical activity prolonged the period of good self-reported health in higher age in two ways: physical activity habits established in early adulthood and onwards were beneficial for self-reported health when entering advanced age. Self-reported health among older adults depended on participation in moderate intensity physical activity after approximately 65 years of age. Additionally, poor self-reported health and being sedentary were independently associated with an increased risk of mortality. The study describes how the most beneficial exercise level varies across age groups in a general population. Although physical activity benefits all, it is likely that physical activity can be particularly critical in certain life situations. The next step should be to examine how physical activity levels vary across different groups, stratified by diagnosis (e.g., diabetes, CVD, cancer), chronic conditions (e.g., pain), and socioeconomic status.

## Supplementary information

**Additional file 1: Supplementary Table 1.** Physical activity level according to age and sex. **Supplementary figure 1.** Activity level according to age and sex.

**Additional file 2: Supplementary Table 2.** Results from the random coefficient proportional odds model with estimates for the sex-specific associations of subject-specific factors with self-reported health.

**Additional File 3 Supplementary Table 3**. Hazard ratio for all-cause death between combinations of categories of hard and light physical activity levels ores as time-dependent covariates.

## Data Availability

The dataset supporting the article findings is available through application directed to the Tromsø Study by following the steps presented on their online page: https://en.uit.no/forskning/forskningsgrupper/sub?p_document_id=453582&sub_id=71247.

## Abbreviations

BMI: Body Mass Index
CC: Complete cases
CONOR-MHI: CONOR Mental Health Index
CVD: Cardiovascular disease
HII: Health Impact Index
HRs: Hazard ratios
HSCL-10: Hopkins Symptoms Checklist
IPW: Inverse probability weighting
OR: Odds ratio
TS: Tromsø Study
WHO: World Health Organization
