# Correction to: A prospective study on the effect of self-reported health and leisure time physical activity on mortality among an ageing population: results from the Tromsø study

**DOI:** 10.1186/s12889-021-10840-7

**Published:** 2021-05-12

**Authors:** Ida Marie Opdal, Lill Sverresdatter Larsen, Laila Arnesdatter Hopstock, Henrik Schirmer, Geir Fagerjord Lorem

**Affiliations:** 1 Department of Psychology, Faculty of Health Sciences, UiT - The Arctic University of Norway, Tromsø, Norway; 2 Department of Health and Care Sciences, Faculty of Health Sciences, UiT - The Arctic University of Norway, Tromsø, Norway; 3 Department of Community Medicine, Faculty of Health Sciences, UiT - The Arctic University of Norway, Tromsø, Norway; 4 Department of Clinical Medicine, Faculty of Health Sciences, UiT - The Arctic University of Norway, Tromsø, Norway; 5 Department of Cardiology, Akershus University Hospital, Lørenskog, Norway; 6 Department of Clinical Medicine, Faculty of Medicine, University of Oslo, Oslo, Norway

**Correction to: BMC Public Health 20, 575 (2020)**

**https://doi.org/10.1186/s12889-020-08681-x**

It was highlighted that in the original article [1] Table 4 was omitted from the published version and Fig. 3 was erroneously included. Fig. 4 and Fig. 5 have been renamed to Fig. 3 and Fig. 4 respectively. This Correction article includes Table 4. The original article has been updated.

**Table 4:** Mortality rates and hazard ratio for all-cause death between categories of physical activity levels in the Tromsø study 1994-2008 until end of follow up on December 31, 2015

	Counts	Died	Hazard ratio (Univariate models)			Hazard ratio (Adjusted model)		
			HR (95% CI)			HR (95% CI)		
**Self-Reported Health**								
Poor	1125	416	5.24 (3.88, 7.06)			2.51 (1.84, 3.42)		
Not so good	8914	2581	3.16 (2.53, 3.94)			2.10 (1.68, 2.64)		
Good	15689	2045	1.64 (1.39, 1.94)			1.35 (1.14, 1.59)		
Very good (ref)	4432	230	1.00			1.00		
**Hard physical activity levels**								
Sedentary	13075	3446	1.69 (1.50, 1.91)			1.32 (1.16, 1.49)		
Some high intensity	9596	891	1.03 (0.90, 1.19)			1.01 (0.88, 1.17)		
Moderate high intensity	6936	587	0.98 (0.85, 1.13)			1.00 (0.86, 1.15)		
Vigorous high intensity	3412	339	1.00			1.00		
**Light physical activity levels**								
None	13075	3446	1.61 (1.50, 1.74)			1.23 (1.13, 1.33)		
<1 hour per week	9596	891	1.20 (1.11, 1.31)			1.02 (0.94, 1.11)		
1-2 hours per week	6936	587	1.04 (0.97, 1.11)			0.97 (0.91, 1.04)		
>3 hours per week	3412	339	1.00			1.00		
**Time Varying Covariates**								
Self-Reported Health			1.016	1.009	1.023	1.009	1.002	1.016

*HR* Hazard ratio, Number of participants = 24831, deaths=5508, Time at risk = 466436 person years

Univariate models: Estimates are controlled for gender and age. SRH was entered as time varying covariate to

The adjusted model: Estimates are controlled for gender, age, comorbid disease, mental health symptoms, cardio-vascular disease risk factors, BMI and smoking habits. SRH was entered as time varying covariate to control for interaction with time
