# Supplementary figures and images for: A prospective study on the effect of self-reported health and leisure time physical activity on mortality among an ageing population: results from the Tromsø study
Source: BMC Public Health. 2020 Apr 28;20:575. doi: 10.1186/s12889-020-08681-x (PMC7189588; doi:10.1186/s12889-020-08681-x)

Supplementary Figure 1. Activity level according to age and sex

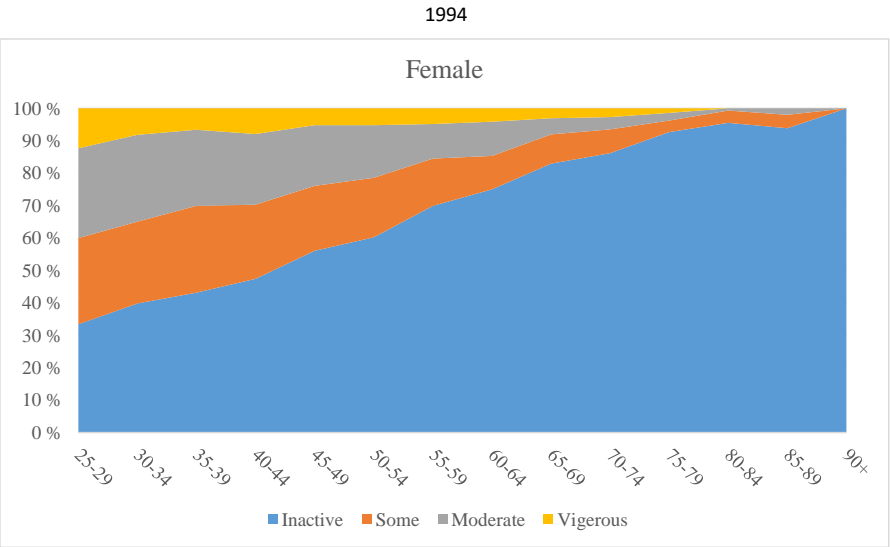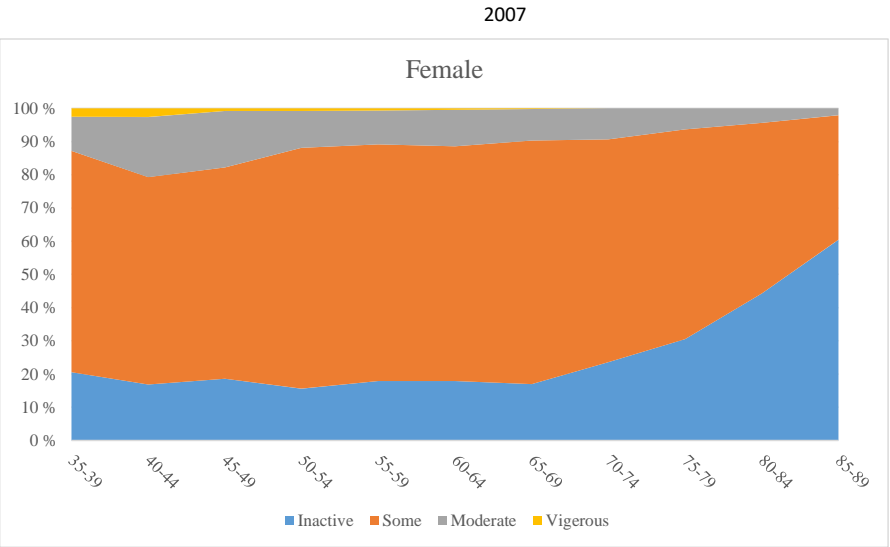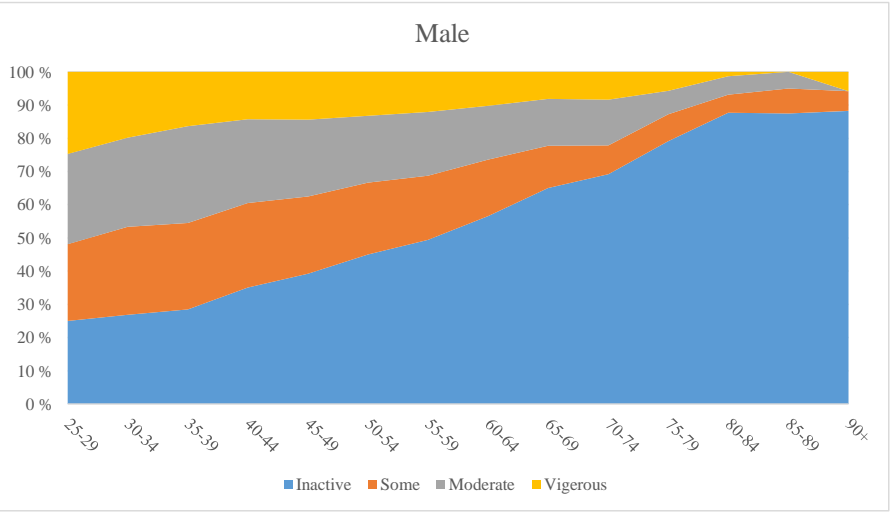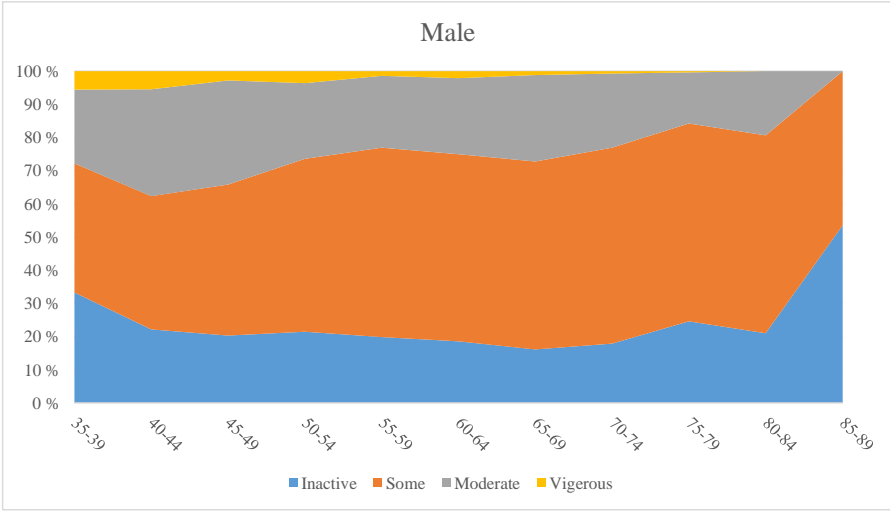

Supplement: Supplementary file 1 — Additional file 1: Supplementary Table 1. Physical activity level according to age and sex. Supplementary figure 1. Activity level according to age and sex. [file 12889_2020_8681_MOESM1_ESM.pdf]
