# Supplementary material for: A prospective study on the effect of self-reported health and leisure time physical activity on mortality among an ageing population: results from the Tromsø study
Source: BMC Public Health. 2020 Apr 28;20:575. doi: 10.1186/s12889-020-08681-x (PMC7189588; doi:10.1186/s12889-020-08681-x)
Supplement: Supplementary file 2 — Additional file 2: Supplementary Table 2. Results from the random coefficient proportional odds model with estimates for the sex-specific associations of subject-specific factors with self-reported health. [file 12889_2020_8681_MOESM2_ESM.pdf]

Supplementary Table 2. Results from the random-coefficient proportional odds model with estimates for the gender specific associations of subject-specific factors on Self-Reported Health.

	Female			Male		
	OR	(95% CI)		OR	(95% CI)	
**Hard physical activity**						
Sedentary (reference)	1,00			1,00		
Some high intensity	1,15	1,03	1,29	1,26	1,11	1,43
Moderate high intensity	1,62	1,40	1,87	2,23	1,92	2,59
Vigorously high intensity	2,00	1,55	2,57	3,31	2,70	4,04
**Light physical activity**						
None (reference)	1,00			1,00		
<1 Hour	1,20	0,96	1,50	0,95	0,77	1,17
1-2 hour	1,39	1,12	1,71	1,26	1,03	1,54
>3 hours	1,62	1,32	1,99	1,29	1,06	1,58
**Gender and age**						
Age (10 year)	0,55	0,52	0,57	0,57	0,53	0,60
Gender	0,89	0,81	0,98	0,88	0,81	0,95

The model is controlled for all covariates
