# Supplementary material for: A prospective study on the effect of self-reported health and leisure time physical activity on mortality among an ageing population: results from the Tromsø study
Source: BMC Public Health. 2020 Apr 28;20:575. doi: 10.1186/s12889-020-08681-x (PMC7189588; doi:10.1186/s12889-020-08681-x)
Supplement: Supplementary file 3 — Additional File 3 Supplementary Table 3. Hazard ratio for all-cause death between combinations of categories of hard and light physical activity levels ores as time-dependent covariates. [file 12889_2020_8681_MOESM3_ESM.pdf]

Supplementary Table 3. Hazard ratio for all-cause death between combinations of categories of hard and light physical activity levels ores as time-dependent covariates.

		Hazard ratio	[95% Conf, Interval]
**Light physical activity levels**			
None		0,97	(0,67, 1,42)
<1 hour per week		1,38	(0,89, 2,13)
1-2 hours per week		1,18	(0,87, 1,62)
>3 hours per week			
**Hard physical activity levels**			
None		1,56	(1,34, 1,83)
Low		1,16	(0,96, 1,40)
Moderate		1,06	(0,88, 1,27)
Vigorous			
**Interaction**			
Light physical activity levels	Hard physical activity levels		
None	*None*	1,57	(1,07, 2,31)
None	*Some*	1,20	(0,62, 2,30)
None	*Moderate*	0,88	(0,50, 1,54)
<1 hour per week	*None*	0,85	(0,54, 1,32)
<1 hour per week	*Some*	0,65	(0,40, 1,07)
<1 hour per week	*Moderate*	0,59	(0,33, 1,03)
1-2 hours per week	*None*	0,87	(0,63, 1,20)
1-2 hours per week	*Some*	0,73	(0,51, 1,04)
1-2 hours per week	*Moderate*	0,81	(0,56, 1,17)

Test statistics: LR chi2(17) = 10633.14, p<0.0001
